# Mesenchymal Stem Cells Engineered to Inhibit Complement-Mediated Damage

**DOI:** 10.1371/journal.pone.0060461

**Published:** 2013-03-26

**Authors:** Melisa A. Soland, Mariana Bego, Evan Colletti, Esmail D. Zanjani, Stephen St. Jeor, Christopher D. Porada, Graça Almeida-Porada

**Affiliations:** 1 Wake Forest Institute for Regenerative Medicine, Wake Forest School of Medicine, Winston-Salem, North Carolina, United States of America; 2 Laboratory of Human Retrovirology, Institut de Recherches Cliniques de Montréal, Montréal, Québec, Canada; 3 Department of Animal Biotechnology, University of Nevada Reno, Reno, Nevada, United States of America; 4 Department of Microbiology and Immunology, School of Medicine, University of Nevada Reno, Reno, Nevada, United States of America; University of Medicine and Dentistry of New Jersey, United States of America

## Abstract

Mesenchymal stem cells (MSC) preferentially migrate to damaged tissues and, due to their immunomodulatory and trophic properties, contribute to tissue repair. Although MSC express molecules, such as membrane cofactor protein (CD46), complement decay-accelerating factor (CD55), and protectin (CD59), which confer protection from complement-mediated lysis, MSC are recruited and activated by anaphylatoxins after transplantation, potentially causing MSC death and limiting therapeutic benefit. We have previously demonstrated that transduction of MSC with a retrovirus encoding HCMV-US proteins resulted in higher levels of MSC engraftment due to decreased HLA-I expression. Here, we investigate whether engineering MSC to express US2 (MSC-US2), US3 (MSC-US3), US6 (MSC-US6), or US11 (MSC-US11) HCMV proteins can alter complement recognition, thereby better protecting MSC from complement attack and lysis. HCMV-US proteins increased MSC CD59 expression at different levels as determined by flow cytometric evaluation of the median fluorescence intensity ratio (MFI). A significant increase in CD59 expression was seen in MSC-US2, MSC-US3, and MSC-US6, but not in MSC-US11. Only MSC-US2 displayed increased expression of CD46, while US2 and US3 proteins were both able to augment the percentage of MSC expressing this molecule. Regardless of the HCMV protein expressed, none changed CD55 MFI; however, expression of US6, US11, and US2 each increased the percentage of MSC that were positive for this molecule. Because US2 protein was the most efficient in up-regulating all three complement regulatory proteins, we used a functional complement-mediated cytotoxicity assay to investigate whether MSC-US2 were protected from complement-mediated lysis. We demonstrated that over-expression of the US2 protein reduced complement lysis by 59.10±12.89% when compared to untransduced MSC. This is the first report, to our knowledge, describing a role of HCMV-US proteins in complement evasion, and our data shows that over-expression of US2 protein on MSC could serve as a strategy to protect these cells from complement lysis.

## Introduction

The complement system, an integral component of the innate defense mechanism, [1] plays an essential role in the inflammatory process, and serves as a critical bridge between the innate and adaptive arms of the immune response [2], [3], [4]. Upon activation, complement proteins function as chemotactic factors and amplifiers of the inflammatory response, promote the destruction of infectious agents, cause cytolysis of damaged cells and tissues, and play an active role in allograft rejection [5], [6].

Mesenchymal stem cells (MSC) express functional receptors for anaphylatoxins C3a and C5a, and these receptors contribute to the recruitment of MSC to sites of injury [7]. In addition to being recruited to sites of complement activation, MSC can also activate the complement system, which results in production of soluble C3a and C5a and deposition of complement-activated molecules on their surfaces [8]. MSC express soluble factor H, the complement regulatory proteins CD46, CD55, and CD59, allowing MSC to be able to inhibit activation of the complement system to a certain extent [8], [9], [10]. Still, in the presence of an activated complement system, these innate mechanisms of protection are insufficient to prevent cellular damage and death [11]. It is possible that despite displaying molecules which confer protection from complement-mediated lysis, MSC are still able to be damaged by the activated complement proteins after being recruited to wound sites, leading to premature cell death. Indeed, recent studies have demonstrated that complement activation is responsible for MSC injury after infusion, and that inhibition of complement activation could be a novel strategy to improve existing MSC-based therapies [11].

Human cytomegalovirus (HCMV) is a ubiquitous pathogen, usually causing an asymptomatic primary infection, remaining latent throughout life [12]. This virus has evolved several strategies to avoid immune system recognition, allowing life-long co-existence with its host [13]. In order to evade the complement system, upon egression from the infected cell, HCMV incorporates the host-encoded complement inhibitor proteins, CD55 and CD59, into its envelope [14]. Furthermore, HCMV has been shown to up-regulate the host-encoded CD55 and CD46 after infection of human fibroblasts [15]. We and others have previously shown that genetically engineering mesenchymal, and other cell populations, with retroviral vectors encoding HCMV proteins US2, US3, US6, and US11 down-modulated HLA-I expression [16], [17], [18], leading to a decrease in allogeneic cytotoxic T lymphocyte (CTL) activation and natural killer cell (NK) killing [19]. In this study, we investigated the role of the HCMV US proteins US2, 3, 6 and 11 in protecting MSC from complement lysis, and demonstrated that different US proteins up-regulate the expression of complement regulatory proteins at different levels, but that overexpression of US2 protein on MSC enhanced the production of all of the complement regulatory molecules expressed on these cells. Furthermore, using a complement lysis assay, we demonstrated that expression of US2 on MSC protected these cells from complement lysis. Therefore, expression of HCMV US proteins on MSC, particularly US2, might constitute a potential strategy by which, in mismatched recipients, one could increase MSC engraftment and/or persistence in the injured tissue in order to release trophic factors.

## Materials and Methods

### Cell lines

Mesenchymal stem cells were purified from fetal liver tissue as previously described [20]. Liver tissue was purchased from Advanced Bioscience Resources (Alameda, CA, USA), homogenized to obtain a single-cell suspension, and Stro-1+ cells were isolated by using anti-Stro-1 antibody (R&D Systems, Minneapolis, MN) and magnetic cell sorting (Miltenyi Biotec, Inc., Auburn, CA) according to manufacturer's guidelines. Stro-1+ cells were then cultured at low density in 0.02% gelatin (Sigma, St Louis MO) on coated flasks using MSCGM™ (Mesenchymal Stem Cell Growth Medium BulletKit® Lonza, Walkersville, Maryland, USA) medium. The RetroPackTM PT67 Packaging Cell Line, purchased from Clontech (Clontech, Mountain View, CA), was used to generate the recombinant retroviral vectors. These studies were performed and approved according to the guidelines from the Office of Human Research Protection at the University of Nevada at Reno.

### Construction of recombinant vectors

Construction of the retroviral vectors used in these studies have been previously reported in detail [19]. Briefly, US2, US3, US6, and US11 coding regions were PCR amplified from a clinical HCMV isolate (kindly provided by Dr. Stephen St. Jeor, University of Nevada Reno, Nevada, USA), introducing *EcoRI* and *XhoI* restriction sites for US3, US6, and US11, and *EcoRI* and *SalI* for US2. The purified PCR products were then ligated into pMSCV-Neo retroviral vector (Clontech, Mountain View, CA) that had been previously digested with *EcoRI* and *XhoI* or *SalI*. These recombinant plasmids were transformed into One Shot Top10 chemically competent cells (Invitrogen, Grand Island, NY), and transformed Top10 cells were selected with ampicillin 50 µg/ml (Sigma, St Louis MO). Positive clones were confirmed by PCR using primers for the US insert, miniprep digestions, and sequencing. For US3, US6, and US11 cloning, digestion was performed with *EcoRI* and *XhoI* restriction enzymes. For US2 cloning, *EcoRI* and *BglII* restriction sites were digested, as US2 cloning generated a SalI/XhoI hybrid restriction site.

### Establishment of retrovirus-producing cell lines

Each US recombinant and an empty plasmid were used to transfect the RetroPackTM PT67 Packaging Cell Line (Clontech, Mountain View, CA) using Lipofectamine 2000 (Gibco-Invitrogen, Carlsbad, CA) according to the manufacturer's instructions. Stable transformants were selected with 500 µg/ml G418 (Invitrogen, Grand Island, NY) at 72 hrs after transfection and for 5 days thereafter. Supernatants were collected and filtered with 0.45 µm low binding protein-syringe filter (Pall Corporation, Port Washington,NY) to eliminate live cells and cell debris.

### Transduction of MSC

Subconfluent cultures of MSC were transduced for 6 hours with filtered collected supernatant containing either US recombinant or empty pMSCVneo retrovirus diluted 1∶1 in serum-free QBSF60 medium (Quality Biological Inc., Derwood, MD) and 8 µg/mL protamine sulfate (Calbiochem, La Jolla, CA). MSCGM™ medium (Mesenchymal Stem Cell Growth Medium BulletKit® Lonza, Walkersville, Maryland, USA) was added after the transduction. At 48 hrs, 500 µg/ml of G418 (Invitrogen, Grand Island, NY) was added and MSC were incubated for 5 days, replacing the selection media every two/three days. Stable transduced cells were analyzed for transgene expression by PCR in order to amplify the US gene and neoR, and were designated as MSC-US2, MSC-US3, MSC-US6 and MSC-US11 according to the HCMV US protein that they expressed, or MSC-E for carrying the empty vector. Primers to amplify neomycin were: F-5′ GTGGAGAGGCTATTCGGCTA3′ and R-5′ CCTTGAGCCTGGCGAACAGT3′. Total RNA was purified from transduced and not transduced MSC by using TRIzol® Reagent with the PureLink™ RNA Micro Kit (Gibco – Invitrogen, Carlsbad, CA). 5 µl of RNA were DNase-treated with RQ1 RNase-Free DNase (Promega, Madison, WI). RT was carried out using the SuperScript III first-strand synthesis system and random primers (Gibco – Invitrogen, Carlsbad, CA) according to the manufacturer's protocol. For PCR, the reaction mixture consisted on 1.5 mM MgCl_2_, 20 mM Tris- HCl (pH 8.3), 50 mM KCl, 0.2 mM dNTP mixture, 0.02 U of Taq DNA recombinant polymerase (Gibco – Invitrogen, Carlsbad, CA), 0.004 µg/µl of each primer and 5 µl of RNA DNase treated. Primers used for cloning and analyzing presence of US genes were the following:

US2 F Eco, 5′ATA**GAATTC**A-ATGAACAATCTCTGGAAAGC3′;

US2R-SalI, 5′ATT**GTCGAC**-TCAGCACACGAAAAACCG3′;

US3F-EcoRI 5′ATA**GAATTC**AA-ATGAAGCCGGTGTTGG3′;

US3R-Xho I 5′GCG**CTCGAG**-TTAAATAAATCGCAGACGG3′

US6F-EcoRI 5′AAG**GAATTC**-ATGGATCTCTTGATACGTCTCGG3′

US6R-Xho I 5′ ATT**CTCGAG**-TCAGGAGCCACAACGTCG3′

US11F-EcoRI 5′ TAA**GAATTC**A-ATGAACCTTGTAATGCTT3′

US11 R-Xho I T5′AT**CTCGAG**-TCACCACTGGTCCG3′.

The amplification conditions were initial denaturation at 94°C (3 minutes), followed by 30 cycles of denaturation at 94°C (45 seconds), annealing (30 seconds) at 55°C, elongation at 72°C (1 minute 30 seconds), and a final elongation of 10 minutes at 72°C. The PCR products were electrophoresed on 1% agarose gel in 1× Tris-acetate-EDTA buffer and visualized by UV transillumination (Bio-spectrum Imaging System; UVP, Inc., Upland, CA) and visionWorksLS software.

### Flow cytometry analysis

MSC, MSC-E MSC-US2, MSC-US3, MSC-US6 or MSC-US11, were analyzed for CD46, CD55 and CD59 protein surface expressions. A total of 1×10^5^ to 1×10^6^ cells/sample detached in 1× PBS with a BD Falcon™ scraper (BD Bioscience Pharmingen, San Diego, CA) were incubated for 15 min at RT with 10 µl mouse anti-human CD59:RPE (AbD Serotec USA, Raleigh, NC), 10 µl FITC mouse anti-human CD46 (BD Bioscience Pharmingen, San Diego, CA), or 10 µl FITC mouse anti-human CD55 (BD Bioscience Pharmingen, San Diego, CA). Stained cells were washed with 0.1% azide in 1× PBS (Sigma, St Louis MO), centrifuged at 1500 rpm for 5 min, and fixed with 1% formaldehyde (Fisher Scientific, Pittsburgh, PA) in BD FACSFlow Sheath Fluid (BD Bioscience Pharmingen, San Diego, CA), and analyzed using a FACSort system (Becton Dickinson, Mountain View, CA, USA) with CELLQUEST software (Becton Dickinson, Mountain View, CA, USA). For each sample, 2×10^4^ cells were acquired. Forward and side-scatter plots were used to exclude dead cells and debris from the histogram analysis plots. Median Fluorescence Intensity ratio (MFI) was obtained by dividing the Median Fluorescence Intensity obtained for a specific marker by the Median Fluorescence Intensity of the isotype control staining for the same cell population. The percentage of positive cells for the corresponding marker was obtained by performing side scatter versus FL1 or FL2.

### Flow cytometric complement-mediated cytotoxicity assay

1.5×10^5^ MSC or MSC-US2 cells were washed, scraped, and incubated with 5 µg/ml human IgM (myeloma) (Rockland, Gilbertsville, PA, USA) whole molecule in 1 ml of MesenCult®-ACF Medium (StemCell, Palo Alto, CA, USA) for 15 min at room temperature (experimental condition). The cells were centrifuged and washed once with MesenCult®-ACF Medium. Rabbit serum complement (Innovative Research Inc, Plymouth, MN, USA) was added at a 1∶4 dilution in MesenCult®-ACF Medium and the cells were incubated for 30 min at 37°C. The cells were centrifuged, washed twice with PBS and incubated at RT in the dark for 15 minutes with 0.1 µM calcein AM and 8 µM Ethidium Homodimer-1 (Molecular Probes- Invitrogen, Carlsbad, CA) in 1 ml of PBS. Following staining, 20,000 cells were counted on a FACSort system with CELLQUEST software (Becton Dickinson) and Flowjo (Tree Star, Inc. Ashland, OR) was used to analyze the results. In addition, a control containing rabbit serum complement in the absence of human IgM was included (control condition). Before running the experimental and control samples, parameters on the FACSort System were set using single color staining of live and ethanol-fixed MSC (dead cells). Specifically, MSC were scraped from culture flasks and stained with 0.1 µM calcein AM (Molecular Probes- Invitrogen) in 1 ml of PBS for 15 minutes at RT in the dark, and analyzed with the FACSort system and CELLQUEST software (Becton Dickinson). The resultant gate was set as the “live cells”. To set the “dead cell” gate, another aliquot of MSC were fixed with 100% ethanol for 10 minutes, washed with PBS, stained with 8 µM Ethidium Homodimer-1 (Molecular Probes- Invitrogen) in 1 ml of PBS for 15 minutes at RT in the dark, and analyzed with the FACSort system and CELLQUEST software (Becton Dickinson). MSC stained with calcein were counted as viable due to the fact that when calcein freely enters in the cell as a non-fluorescent molecule, it is modified by cell estearases into a fluorescent compound which after excitation at 483 nm emits at 535 nm. Ethidium homodimer-1 is only able to enter cells with membrane damage, i.e., dead or dying cells. Following incorporation into the cell and excitation at 485 nm, this compound emits at 650 nm. Due to the differences in emission spectra between calcein and Ethidium homodimer-1, these compounds can be used simultaneously and allow one to clearly distinguish between live and dead cells. Double-positive cells were counted as having membrane damage, and double-negative cells were excluded from the analysis. The percentage of cytotoxicity was calculated as follows: [(percent of dead cells under experimental conditions – percent of spontaneously dead cells)/(100 – spontaneously dead)]*100 [21]. The percentage of spontaneously dead cells was determined by incubating the cells with 5 µg/ml human IgM (myeloma) whole molecule in MesenCult®-ACF Medium for 15 min at room temperature without adding rabbit serum complement.

### Statistical analysis

Experiments were independently repeated at least three times. Results were presented as mean ± SEM. Unpaired two-tailed student T test was used to analyze the statistical significance of the results obtained with the Flow cytometric complement-mediated cytotoxicity assay.

For the analysis of CD59 surface levels, to compare MFI ratio and percentage of CD59 positive cells, one-way analysis of variance (ANOVA) was performed. After confirming a statistical difference among the groups, Fisher's least significant difference (LSD) test analysis was performed as a post-doc test due to the unequal number of samples between groups. In the case of analysis of CD46 and CD55 MFI ratio on MSC as well as percentage of positive cells, one-way analysis of variance (ANOVA) was performed followed by Tukey's honestly significant difference (HSD) as the post-hoc test due to equal number of samples among the groups.

For all analyses, a p value <0.05 was considered to be statistically significant.

## Results

### Up-regulation of CD59 expression by US HCMV proteins

**Figure 1:**
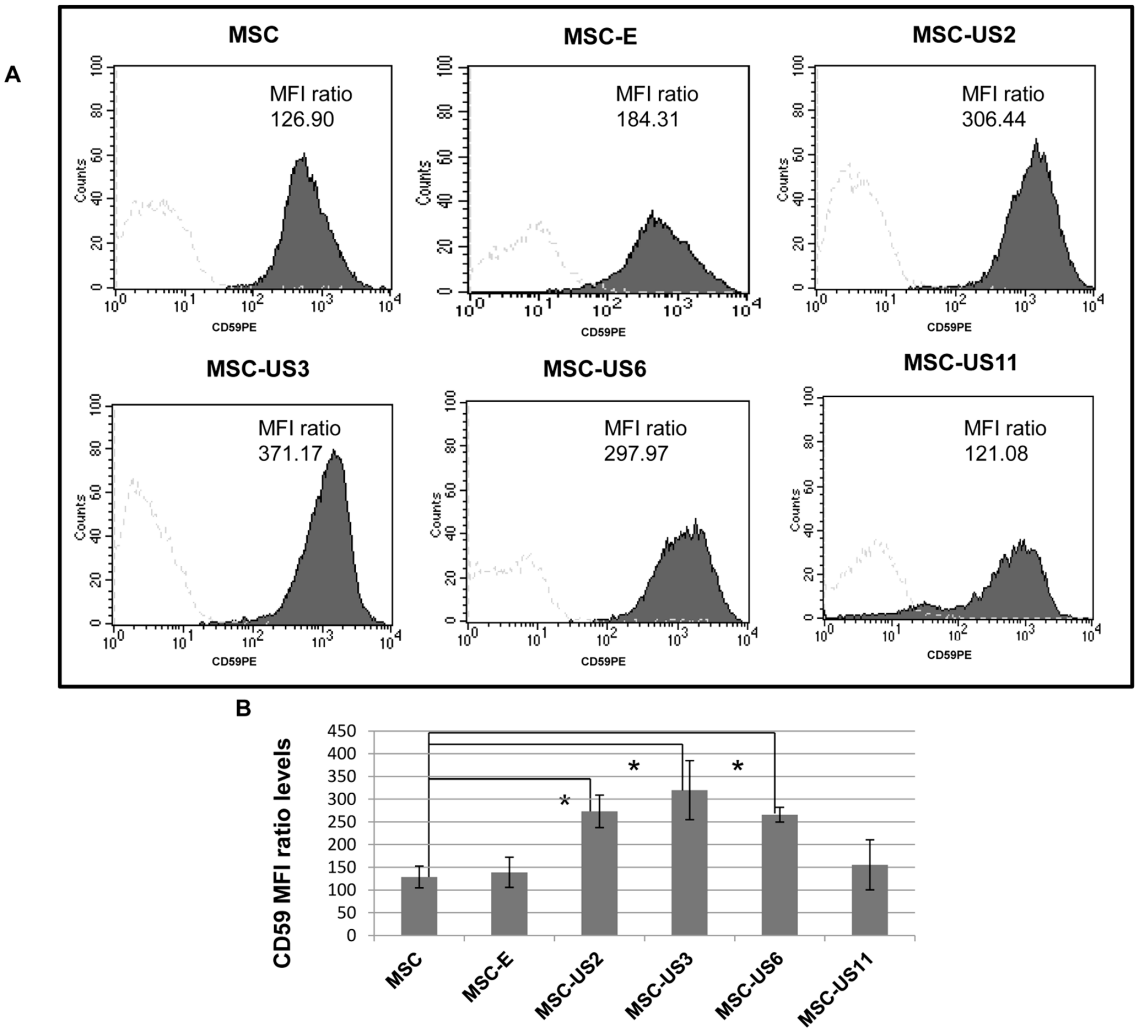
Up-regulation of CD59 surface expression on MSC by HCMV US proteins. (A) MSC, MSC-E, MSC-US2, MSC-US3, MSC-US6, and MSC-US11 were analyzed by flow cytometry for expression of CD59. Each panel depicts representative data of at least three independent experiments. Black filled histograms correspond to different MSC cell populations stained with antibody against CD59, and unfilled histograms are the corresponding isotype control staining. The MFI ratio for each MSC cell line was obtained by performing the following calculation: MFI ratio  =  (Median Fluorescence Intensity for CD59/Median Fluorescence Intensity for isotype control). (B) MFI ratio for each MSC population is shown. The results represent the mean ± SEM from at least three independent experiments. * indicates p<0.05 when comparing MSC-US cells with non-transduced MSC.

It has previously been shown that an increase in cell surface expression of the complement regulatory protein CD59 will confer protection against complement lysis [22]. In order to investigate the effect of US proteins on the expression of CD59, we transduced MSC with the different MSCVneo retroviruses expressing US2, US3, US6 or US11, and used, as a control, both MSC non-transduced (MSC), or transduced with the empty MSCVneo retrovirus expressing only the NeoR gene (MSC-E). Flow cytometric analysis was then used to analyze the surface expression of CD59 on US transduced- and non-transduced MSC as described in the Material and Methods section. Figure 1A depicts representative results obtained from at least 3 independent experiments. Transduction of MSC with pMSCVneo (MSC-E) did not significantly change CD59 median fluorescence intensity (MFI ratio levels: 138.93±33.20) or the percentage of CD59 positive cells (92.38±2.47) when compared to non-transduced MSC (MFI ratio: 128.80±23.94; percentage of CD59 positive cells: 92.64±3.53) (p>0.05) (Figure 1B). Moreover, MSC-US11 did not produce a significant increase in CD59 expression, when compared with untransduced MSC or MSC-E, as determined by MFI ratio: MSC-US11 MFI ratio: 155.49±54.94 or percentage of CD59 positive cells: 93.92±2.30% (p>0.05). However, expression of US2, US3, or US6 proteins significantly increased the levels of CD59 expression, as measured by a change in MFI ratio and percentage of CD59 positive cells, when compared to untransduced MSC and MSC-E (p<0.05). The MFI ratio of CD59 on MSC-US2 was 273.19±35.77, that of MSC-US3 was 319.82±64.87, and on MSC-US6 265.77±16.22. Moreover, 97.27±1.26% of MSC-US2, 96.80±1.47% of MSC-US3 and 96.87±0.69% of MSC-US6 cells were positive for CD59 surface marker.

### US HCMV proteins increase CD46 protein expression

**Figure 2:**
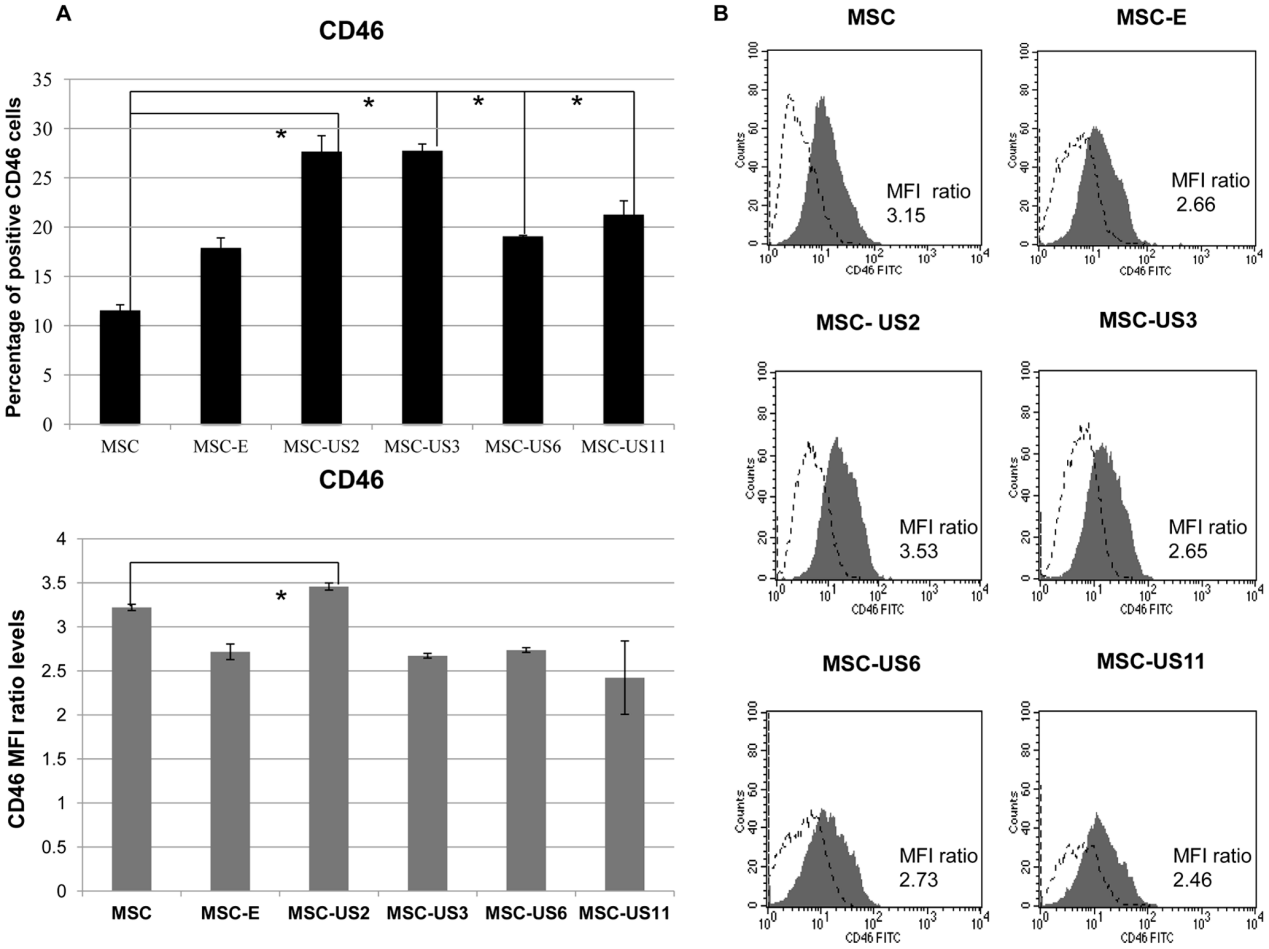
Up-regulation of CD46 surface expression on MSC by HCMV US proteins. (A) MSC, MSC-E, MSC-US2, MSC-US3, MSC-US6, and MSC-US11 were analyzed by flow cytometry for CD46 expression. Top- Percentage of CD46 positive cells for each MSC population. Bottom- MFI ratio for CD46 expression on each MSC population. MFI ratio  =  (Median Fluorescence Intensity for CD46/Median Fluorescence Intensity for isotype control). The results represent the mean ± SEM from at least three independent experiments. * indicates p<0.05 when comparing MSC-US cells with non-transduced MSC. 0(B) Each panel depicts representative data of at least three independent experiments. Black filled histograms correspond to different MSC cell populations stained with antibody against CD46 and unfilled histograms are the staining with the corresponding isotype control.

Next we studied the expression of membrane cofactor protein (MCP), or CD46, another membrane-bound complement regulatory molecule. This protein protects the host cell from complement damage by inactivating the complement components C3b and C4b [23]. Flow cytometric analysis demonstrated that, before transduction, 11.56±0.57% of MSC expressed CD46, and had a MFI ratio of 3.22±0.03, and that transduction with the empty vector, MSCVneo, resulted in a slight increase in the percentage of cells expressing CD46, 17.90±1.01%, (p<0.05) but a decrease in overall expression of CD46: MFI ratio of 2.72±0.09 (p<0.05), (Figure 2A).

MSC-US6 displayed higher percentage of CD46 positive cells when compared to MSC, but this increase was not statistically significant when compared with MSC-E (p>0.05): 19.07±0.1% of MSC-US6 were positive for CD46 (MFI ratio: 2.73±0.03). In contrast, a significant increase in the percentage of CD46 positive cells was found when US11 (21.28±1.38) or even more markedly US3 (27.76±0.68%), was expressed on MSC (p<0.05), but the MFI ratio of these cells (MSC-US3: 2.67±0.03 and for MSC-US11: 2.42±0.42) remained similar to those of MSC-E (p>0.05). MSC-US2 displayed the highest increase in both the percentage of CD46 positive cells (27.67±1.61%)(p<0.05) and the MFI ratio (MFI ratio: 3.46±0.04)(p<0.05). These results showed that expression of US3 and to an even greatest extend US2 on MSC, resulted in efficient up-regulation of CD46 expression on MSC. Figure 2B depicts a representative result from three independent experiments indicating the CD46 MFI ratio levels, obtained in this particular experiment (black filled histogram) and isotype control staining (unfilled histogram).

### CD55 is also up-regulated by expression of US HCMV proteins

**Figure 3:**
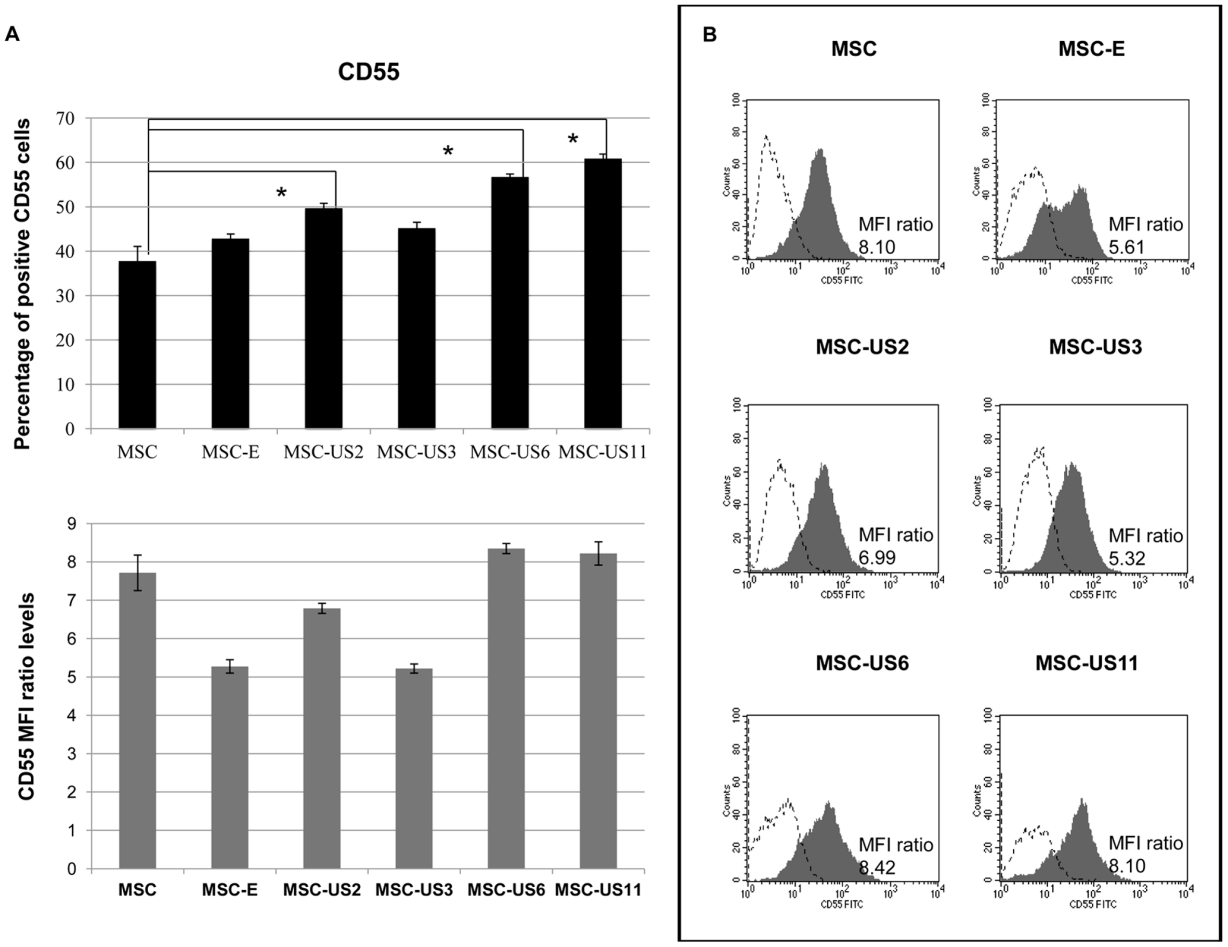
Up-regulation of CD55 surface expression on MSC by HCMV US proteins. (A) MSC, MSC-E, MSC-US2, MSC-US3, MSC-US6, and MSC-US11 were analyzed for CD55 surface expression by flow cytometry. Top- Percentage of CD55 positive cells for each MSC population. Bottom- MFI ratio for CD55 expression on different MSC populations. MFI ratio  =  Median Fluorescence Intensity for CD55/Median Fluorescence Intensity for isotype control. The results represent the mean ± SEM from at least three independent experiments. * indicates p<0.05 when comparing MSC-US cells with non-transduced MSC. (B) Each panel depicts representative data of at least three independent experiments. Black filled histograms correspond to each MSC cell population stained with antibody against CD55 and unfilled histograms correspond to isotype staining.

Complement decay-accelerating factor (DAF), also known as CD55 prevents the assembly of the C3 as well as C5 convertases and blocks the formation of the membrane attack complex [24]. This molecule is expressed constitutively in 37.79±3.30% of MSC (MFI ratio: 7.71±0.46). Transduction of MSC with a retroviral vector encoding only the neoR gene, (MSC-E) did not change the percentage of CD55 positive cells but reduced the MFI ratio; MSC-E: 42.84±1.03% (p>0.05), MFI ratio: 5.27±0.17 (p<0.05). The percentage of MSC-US3 positive for CD55 was 45.21±1.31% and displayed a MFI ratio of 5.22±0.12. Therefore, expression of US3 on MSC resulted in a pattern of CD55 expression similar to that of MSC-E. By contrast, MSC-US2 exhibited a higher increase in the percentage of CD55 positive cells (49.70±1.08%; p<0.05) when compared to MSC or MSC-E (p<0.05), and an increased MFI ratio when compared to MSC-E levels (MFI ratio: 6.79±0.13; p<0.05). Amongst the US HCMV proteins tested, however US6 and US11 were the most effective at enhancing the percentage of positive CD55 cells when compared to non-transduced MSC (p<0.05) or MSC-E (p<0.05). The percentage of MSC-US6 and US11 positive cells for CD55 were 56.72±0.66% and 60.86±1.03% respectively (Figure 3A). Importantly, the MFI ratio for MSC-US6 and MSC-US11 remained similar to that of non-transduced MSC, and was, respectively, 8.35±0.13 and 8.22±0.30. Figure 3B depicts one representative result from three independent experiments, indicating the MFI ratio levels for CD55 staining (black filled histogram) and isotype control staining (unfilled histogram).

### Reduction of complement lysis by stable expression of US2 HCMV protein on MSC

**Figure 4:**
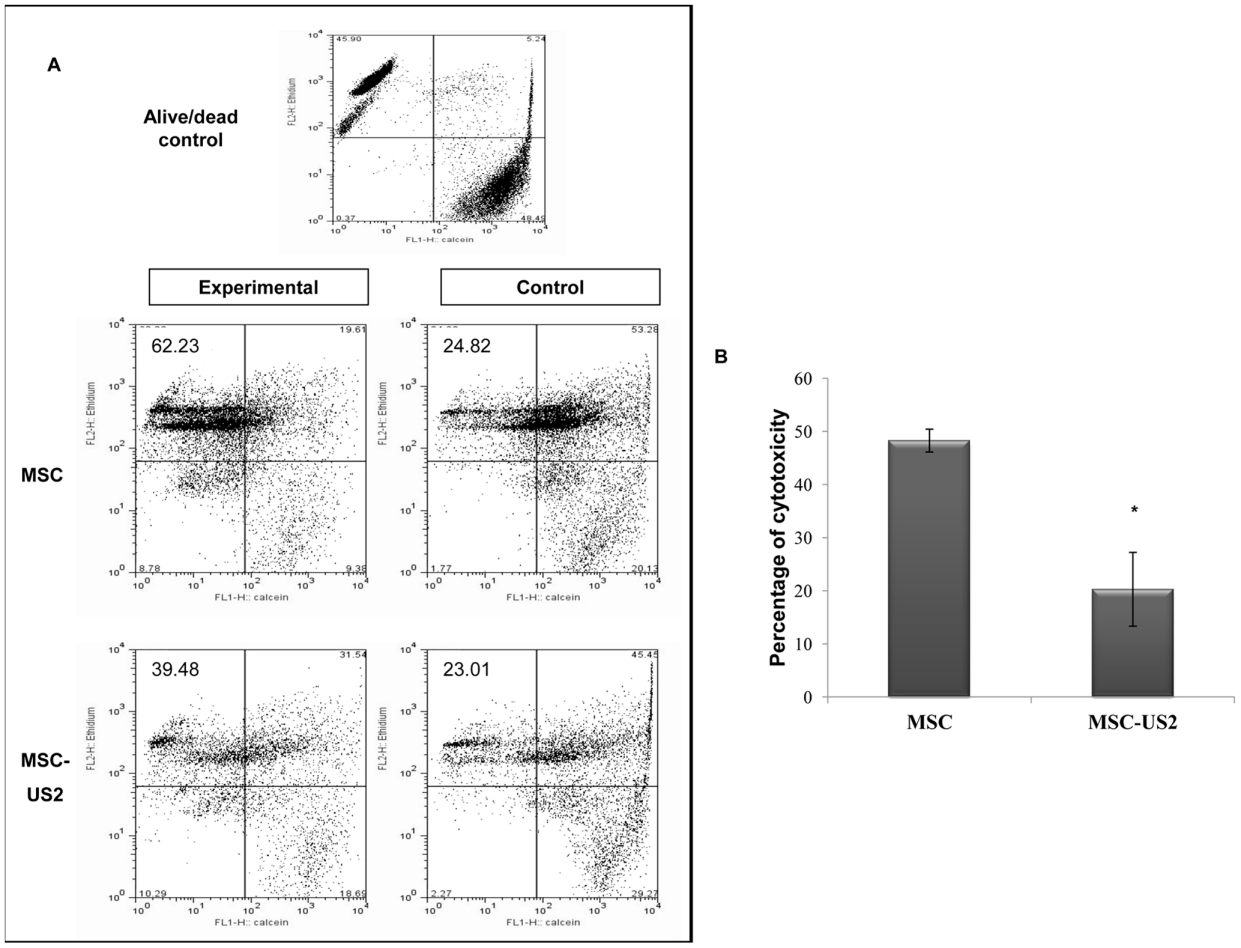
Effect of overexpression of HCMV US2 protein on MSC in a complement-mediated cytotoxicity assay. (A) The results depict representative data from a flow cytometry-based complement-mediated cytotoxicity assay from at least 3 independent experiments. The assay was performed in the presence of human IgM and rabbit serum complement (experimental) or in the presence of human IgM and absence of the rabbit serum complement (control). MSC stained with calcein were counted as viable, cells stained with Ethidium homodimer-1 were counted as dead, and cells doubly positive were counted as having membrane damage. Doubly negative cells were excluded from the analysis. A live/dead control (top) was used in order to set up the quadrant gates for alive, dead, and membrane-damaged populations and to set parameters for compensation between the 2 fluorescent channels. The experimental assay provided the percentage of dead cells under experimental conditions. The control assay provided the percentage of spontaneously dead cells. The same conditions were applied for non-transduced MSC and MSC-US2. (B) The percentage of cytotoxicity for each cell line was calculated as follows: (percent of dead cells under experimental conditions – percent of spontaneously dead cells)/(100 – spontaneously dead)*100. The percentage of experimental or spontaneously dead cells was determined by calculating the percentage of cells that were positive for Ethidium homodimer-1, and negative for calcein. The results depict the mean ± SEM of three independent experiments. * indicates p<0.05.

When compared to other US HCMV proteins, US2 protein appeared to exert the broader effect on modulating expression of complement regulatory molecules, since it was either the most, or at least equally, efficient at up-regulating the MFI ratio and/or the percentage of cells expressing CD46, CD55, and CD59 molecules. Therefore, MSC-US2 was chosen to be further tested, using a functional complement-lysing assay, to investigate the role of this US HCMV protein in protection from complement lysis. Figure 4A shows the results of a representative experiment (n = 3) depicting the flow cytometry-based complement-mediated cytotoxicity assay, performed as described in detail in the Materials and Methods section. MSC were evaluated in the presence of IgM and rabbit serum complement (experimental) or in the presence of IgM and absence of the rabbit serum complement (control). The same experimental and control conditions were used using MSC-US2. The percentage of cytotoxicity was calculated for both cell populations according to the formula described in the Material and Methods section. Complement cytotoxicity on control MSC was 48.27±2.16% but expression of US2 on MSC reduced the percentage of cytotoxicity to 20.27±6.94%, a 59.10±12.89% reduction in complement lysis (p<0.05) (Figure 4B).

## Discussion

The efficacy of allogeneic MSC as therapeutic tools has been reported in numerous studies, (reviewed in [25], [26], [27]); however, controversy still remains whether these cells, upon transplantation, are able to elicit an immune response in the recipient, lessening their therapeutic potential when compared with their autologous counterpart. Studies showed that some of the factors responsible for the failure of donor MSC to engraft include the mismatch of HLA class I molecules between the donor and the recipient [28], [29] and activation and damage by the complement system [11]. Although MSC display on their surface proteins that inhibit complement system activation such as CD59, CD46, CD55 [8], [10] and inhibitor factor H, [9] the complement system still is able to recognize and injure MSC after their infusion [11].

Our results are in agreement with these prior studies showing that MSC constitutively express high levels of CD59, while CD46 and CD55 are so to a lesser degree. Moreover, we show for the first time that engineering MSC to express US2, US3, US6, or US11 HCMV proteins alters the expression of these regulatory complement recognition molecules and protects MSC from complement attack and lysis.

Overexpression of US2, US3, and US6 (but not US11) HCMV proteins, significantly increased CD59 surface molecule levels and the percentage of CD59 positive cells. US6 and US11 proteins did not alter either the expression or the percentage of cells positive for CD46, while US3 induced only an increase in the percentage of MSC that were CD46 positive. However, US2 protein induced the highest increase in the percentage of CD46 positive cells, and in the level of expression on a per cell basis. The US6 and US11 proteins effectively increased the percentage of MSC positive for CD55 but not its intensity of expression, whereas US3 protein left the pattern of CD55 expression on MSC unaltered. By contrast, MSC-US2 exhibited an increase in both the percentage of CD55 positive cells and MFI ratio. Overall, these results demonstrated that expression of US2 HCMV protein on MSC had the ability to increase expression of all three surface molecules MSC produced for protection against complement activation. Although we did not investigate the mechanism by which US2 was able to alter the expression of CD46, CD55 and CD59, it is possible that the disruption and accumulation of HLA-I complexes in the Endoplasmic Reticulum (ER) caused by over expression of US2 leads to ER stress and consequent increase in the levels of NF-κB [30]. Since CD46, CD55 and CD59 are all known to be induced by NF-κB dependent pathways, this could provide a possible explanation for your findings[31]

To establish the functional significance of the altered expression of CD46, CD55 and CD59 by US2 we performed a FACS-based complement-lysing assay, and demonstrated a 59.10±12.89% reduction in complement lysis in MSC expressing US2 protein, when compared to the control.

While recent studies showed that MSC were able to trigger C3 binding to their surface, and higher C3 binding capacity was correlated with higher suppression of PBMC- proliferation [8], we have also recently shown that expression of US proteins, including US2, reduces HLA-I expression and consequently decreases peripheral blood mononuclear cell recognition of MSC [19]. Furthermore, although MSC-US2 expressed reduced levels of HLA-I expression, they were no more susceptible to NK killing than untransduced MSC [19] due to high expression of CD55 molecules [32]. This recent work, combined with the present findings, show that expression of US2 HCMV on MSC provides multiple layers of protection to these cells, decreasing not only peripheral blood mononuclear cell proliferation, but also lysis of MSC by NK cells and by the complement system. In conclusion our data suggests that use of these genetically-engineered MSC, mainly MSC-US2, would have an enhanced advantage in the presence of an exacerbated inflammatory microenvironment and/or in an allogeneic transplantation setting, resulting in decreased rejection by the immune system, extending the time in survival and thereby enhancing their therapeutic potential.
